# HFR1 Is Crucial for Transcriptome Regulation in the Cryptochrome 1-Mediated Early Response to Blue Light in *Arabidopsis thaliana*

**DOI:** 10.1371/journal.pone.0003563

**Published:** 2008-10-30

**Authors:** Xiao-Ning Zhang, Yingjie Wu, John W. Tobias, Brian P. Brunk, Gerald F. Deitzer, Dongmei Liu

**Affiliations:** 1 Plant Science Institute, Department of Biology, University of Pennsylvania, Philadelphia, Pennsylvania, United States of America; 2 Center for Bioinformatics, University of Pennsylvania, Philadelphia, Pennsylvania, United States of America; 3 Department of Plant Science and Landscape Architecture, University of Maryland, College Park, Maryland, United States of America; University of Massachusetts Amherst, United States of America

## Abstract

Cryptochromes are blue light photoreceptors involved in development and circadian clock regulation. They are found in both eukaryotes and prokaryotes as light sensors. Long Hypocotyl in Far-Red 1 (HFR1) has been identified as a positive regulator and a possible transcription factor in both blue and far-red light signaling in plants. However, the gene targets that are regulated by HFR1 in cryptochrome 1 (cry1)-mediated blue light signaling have not been globally addressed. We examined the transcriptome profiles in a cry1- and HFR1-dependent manner in response to 1 hour of blue light. Strikingly, more than 70% of the genes induced by blue light in an HFR1-dependent manner were dependent on cry1, and *vice versa*. High overrepresentation of W-boxes and OCS elements were found in these genes, indicating that this strong cry1 and HFR1 co-regulation on gene expression is possibly through these two *cis*-elements. We also found that cry1 was required for maintaining the HFR1 protein level in blue light, and that the HFR1 protein level is strongly correlated with the global gene expression pattern. In summary, HFR1, which is fine-tuned by cry1, is crucial for regulating global gene expression in cry1-mediated early blue light signaling, especially for the function of genes containing W-boxes and OCS elements.

## Introduction

Light is not only an energy source for photosynthesis, it is also an important signal for plant development. Two different groups of photoreceptors have been intensively studied – the red/far-red light photoreceptors phytochromes (phyA-E) and the blue/UVA light receptors cryptochromes (cry1, cry2, cry-DASH) and phototropins (phot1 and phot2). Cryptochromes are found in plants, animals and microbes, mediating a variety of blue light-dependent responses including growth, development and the entrainment of circadian rhythms [1]–[4]. The *cry1* null mutant plants exhibit pleiotropic phenotypes including elongated hypocotyls, small and unopened cotyledons and shortened roots [1]; indicating that multiple downstream pathways are regulated by cry1 signaling. As more and more downstream regulators have been identified by genetic screening [5]–[12], HFR1, also known as REP1 and RSF1, was defined as a positive downstream factor integrating information from both cry1 and phyA [6].

HFR1 was originally identified based on its role in inhibition of hypocotyl elongation, cotyledon expansion, modulation of gravitropic growth, and induction of certain light-regulated gene expression in phyA signaling [13]–[15]. The *HFR1* gene encodes a light-inducible, transcription regulator containing an atypical basic helix-loop-helix motif. Although it has been located in the nucleus, there is still no evidence showing that HFR1 binds to DNA [13]. Overexpressing the C-terminus of HFR1 constitutively activates a subset of photomorphogenic responses in darkness, far-red and white light. In addition, the inhibition of hypocotyl elongation by HFR1 also depends on phyA, FHY1 and FHY3 under far-red light, or phyB under red light, respectively [16]; suggesting that HFR1 is involved in a complex regulatory network in *Arabidopsis*. In addition to its role in phyA signaling in far-red light, HFR1 is also a component of cry1-mediated blue light signaling [6]. Mutant *hfr1* alleles have reduced de-etiolation responses under blue light. Unlike *hy5*, de-etiolation in *hfr1* mutants is particularly significant under high fluence rates. But it is still unknown how HFR1 identifies its downstream targets and what they are. Although cry1 is the predominant photoreceptor in high fluence blue light, other photoreceptors participate as well. For example, HFR1's function in anthocyanin accumulation is most likely to be affected by a complex combination of cry1, cry2 and phyA [6]. It is still a puzzle how HFR1 integrates different light signals and distinguishes them from each other.

Recent genetic and biochemical studies have found that HFR1 is posttranslationally regulated by 26S proteasome-mediated degradation. Constitutively Photomorphogenic 1 (COP1), an E3 ligase, can physically interact with the N-terminus of HFR1 to mediate ubiquitination of phosphorylated HFR1, which may subject HFR1 to degradation [17], [18]. Similar posttranslational regulation has been found in other factors in photomorphogenesis as well [7], [19], [20].

To further our understanding on the role of HFR1 in cry1-mediated blue light signaling, we used microarray techniques to compare the transcriptome profiling among wild-type (WT), *cry1* and *hfr1* mutants after 1 h blue light treatments. HFR1 and cry1 protein levels were examined under the same conditions as well. Interestingly, we found a close relationship between cry1 and HFR1 on the induction of gene expression. cry1 depends on HFR1 to turn on certain genes, and HFR1 requires cry1 to sustain its protein stability. Using computational methods, we also propose that HFR1 may elevate downstream gene expression through W-boxes and OCS elements, indicating that a subset of genes, having either of these *cis*-elements, may be responsive to cry1-mediated early blue light signaling.

## Results

### *hfr1-23* mutant isolation and identification

**Figure 1:**
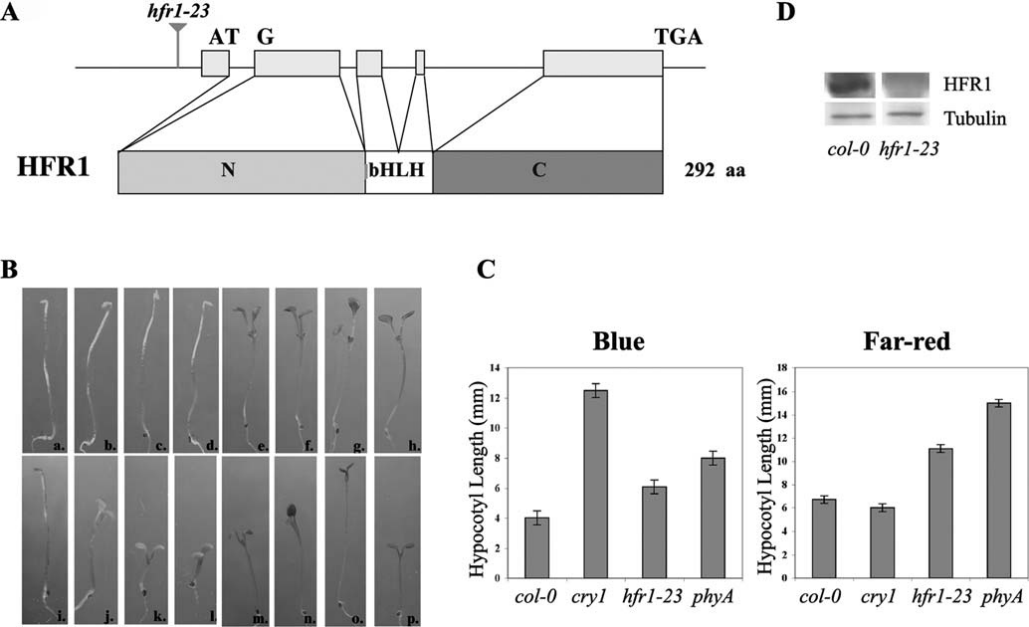
Characterization of *hfr1-23* mutants. (A) T-DNA insertion identified in *HFR1* gene. A schematic diagram representing *HFR1* gene with its intron/exon structrure, the encoded protein, and the location of T-DNA insertion in *hfr1-23* mutant. N, bHLH and C represent the N-terminus, basic helix-loop-helix domain and C-terminus of HFR1 protein, respectively. (B) Seven-day-old Arabidopsis seedlings of *phyA*, *hfr1*, *cry1* and WT in different light conditions. Seedlings from left to right are *phyA*, *hfr1*, *cry1* and WT. a–d: Seedlings grown in the dark; e–h: Seedlings grown in red light (30 *µmol m^−2^ s^−1^*); i–l: Seedlings grown in far-red light (10.3 *µmol m^−2^ s^−1^*); m–q: Seedlings grown in blue light (30 *µmol m^−2^ s^−1^*). (C) Hypocotyl elongation of WT, *phyA*, *cry1* and *hfr1* seedlings in blue (15 *µmol m^−2^ s^−1^*) and far-red light (10.3 *µ mol m^−2^ s^−1^*). All seedlings were grown in continuous light for 4 days before measurement. 20 seedlings were used in each sample. Error bars represent standard deviations. (D) HFR1 protein level in *hfr1-23* mutants and *col-0*. β-Tubulin was used as control. The seedlings were grown in blue light (50 *µmol m^−2^ s^−1^*) for 3 days.

Genetic screening was conducted among the T-DNA mutant pools (CS31087). One mutant showing longer hypocotyls than WT, in both blue and far-red light, was selected and crossed back to WT three times. A T-DNA insertion, located at −276 bp upstream of the start codon in the *HFR1* gene, was found linked to the long hypocotyl phenotype (Figure 1A). HFR1 protein was undetectable in *hfr1-23* mutants, while it was strongly accumulated in WT (Figure 1D). This suggested that *hfr1-23* was a null mutant.

The *hfr1-23* seedlings had the same phenotype under blue and far-red light as described earlier for other alleles [13]–[15]. They were deficient in their inhibition of hypocotyl elongation and cotyledon opening compared to WT seedlings, but the phenotype was less severe than *cry1* mutants in blue light and *phyA* mutants in far-red light, respectively. They were indistinguishable from the WT under either red light or darkness (Figure 1B and 1C). Given all the evidence above, we concluded that *hfr1-23* is a new allele of *hfr1* and it will be referred to *hfr1* in the rest of the text for convenience.

### Transcriptome profiling of WT, *cry1* and *hfr1* mutants

**Figure 2:**
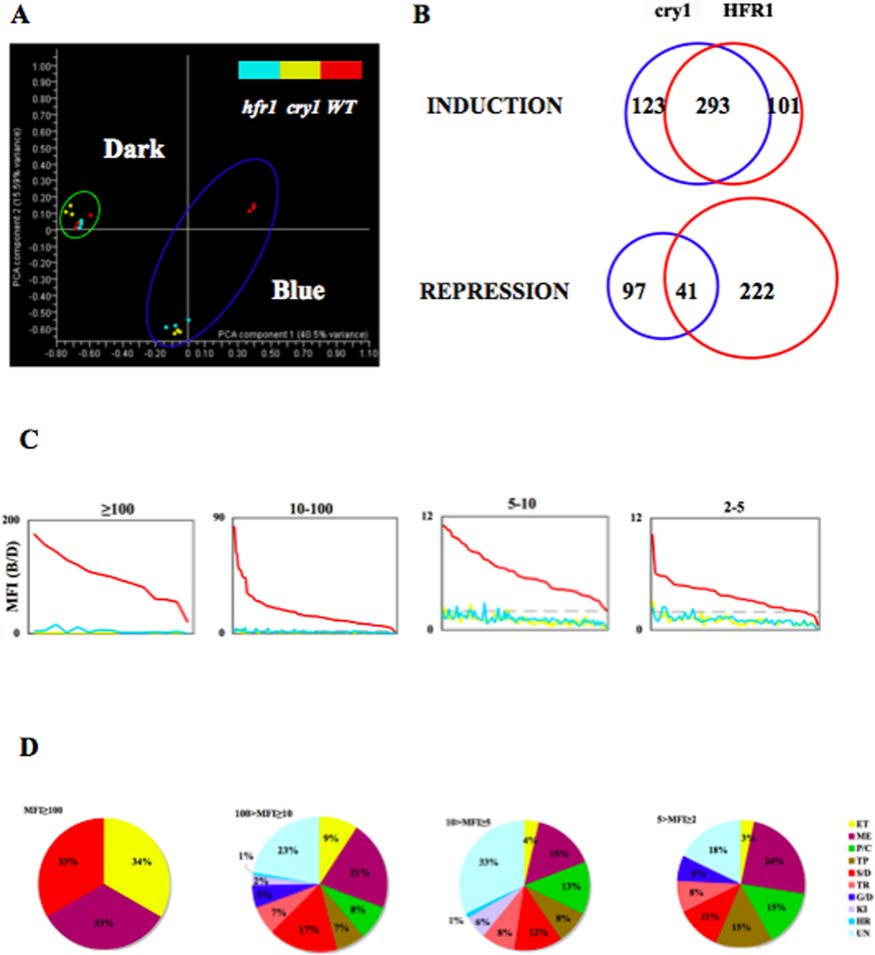
Gene expression profiling *in* WT, *cry1 and hfr1* mutants in the dark and 1 h blue light (50 *µmol m^−2^ s^−1^*). (A) Evaluation of the profile by Principle Component Analysis (PCA). All genes on ATH1 chips were used to generate the PCA plot. WT, *cry1* and *hfr1* represented the global gene expression in WT, *cry1B104* and *hfr1-23* mutants. Three biological replicates in the same genetic background for each treatment were colored differently (red: WT, yellow: *cry1* and blue: *hfr1*). All samples in dark treatment were grouped in a green circle. All samples in blue light treatment were shown in a blue circle. (B) Genes regulated by blue light in a cry1- and HFR1-dependent manner. INDUCTION: Genes induced by blue light and dependent on cry1 and HFR1; REPRESSION: genes repressed by blue light and dependant on cry1 and HFR1. Genes regulated by blue light in cry1-dependent manner (blue circle) were determined by 2-way ANOVA with p-value lower than the false discovery rate (3.87E-05) in the comparison of WT/*cry1*; genes regulated by blue light in HFR1-dependent manner (red circle) were determined by 2-way ANOVA with p-values lower than the false discovery rate (4.62E-05) in WT/*hfr1* comparison. All genes were applied as universe. Numbers in each portion showed the gene distribution. (C) Blue light induction of genes in WT, *cry1* and *hfr1* mutants. 293 blue light-induced genes, dependant on both cry1 and HFR1 from (B), were divided into four groups based on their mean fold induction (MFI) of cry1 in blue light (MFI≥100, 10–100, 5–10 and 2–5). Genes in each group were plotted in rank order of their relative response to 1 h blue light compared to the dark treatment. Curves represented MFI values for WT (red), *cry1* (yellow) and *hfr1* (blue) mutants. (D) Distribution of genes induced more than 2-fold by cry1 in 1 h blue light among functional categories, shown as a percentage of the total annotated genes within each group. ET: electron transport; ME: metabolism; P/C: photosysthesis/chloroplast; TP: transport; S/D: stress/defense; TR: transcription; G/D: growth/development; KI: protein kinases; HR: hormones; UN: unknown.

To understand the role that HFR1 may play in the cry1 pathway, we used Affymatrix GeneChip ATH1 to perform a microarray analysis. The *cry1*, *hfr1* mutants and WT seedlings were grown in the dark for 4 days and then exposed to 50 *µmol m^−2^ s^−1^* of blue light for 1 hour, or allowed to remain in the dark, for further transcriptome comparison. Three biological replicates were prepared for WT, *cry1* and *hfr1* individually for hybridization, scanning of the chips and further statistical analysis. All 22810 genes presented on the chips were used for statistical evaluation (Data S1). Principal Component Analysis (PCA) showed that three replicates from each WT/mutant comparison were grouped together, and the variation within the triplicates was significantly less than the variation among WT and the two mutants; suggesting that the data were reproducible. In the dark treatment, both mutants had very similar profiles to WT. When exposed to 1 h blue light, *cry1* and *hfr1* mutants became strikingly similar to each other, while WT was much more distinguishable from both mutants (Figure 2A). This suggested that the transcriptome of both the *cry1* and *hfr1* mutants responded to 1 h of blue light in a similar manner and that they were significantly different from WT.

All samples were defined by their genotype (G: WT, *cry1*, *hfr1*) and by the light treatment (L: dark, blue), which were used as two independent factors applied in 2-way ANOVA analysis. In each pair-wise comparison (WT/*cry1* and WT/*hfr1*), all genes were calculated for the variance of G, L and the interaction of G*L. The significance of the variance was quantified as the p-value. Genes with p-values lower than the false discovery rate for interaction of G*L were selected as regulated by blue light and dependent on the genotype in each comparison. The magnitude of the contribution of cry1 or HFR1 on each gene in the blue light response was quantified by the mean fold change for the induction or repression of each gene. A fold change of 1.0 represents no contribution by cry1 or HFR1, respectively. The fold change of all of the candidates taken into account was expected to be higher than 2.0 (including 2.0), which represents a significant effect of cry1 or HFR1, respectively. In each pair-wise comparison, all candidates regulated by the interaction of G and L with more than a 2 fold change (including 2 fold) in blue light were selected to generate gene lists representing genes induced or repressed by blue light and also dependent on the genotype. Strikingly, 74% (293 of 398) of the genes that were identified as blue light-induced, and dependent on HFR1, were also found to be dependent on cry1. These 293 genes were 70% of the 416 genes induced by blue light and dependent on cry1. However, only 16% (41 of 263) of the genes that were blue light-repressed, and dependent on HFR1, were found to be dependent on cry1, which is 30% of the 138 genes that were repressed by blue light and dependent on cry1 (Figure 2B, Data S6). Neither the quantity (41) nor the mean magnitude of repression (2–14.54 fold) of these repressed genes is comparable to the induced genes (Data S6). These 41 repressed genes are a much smaller proportion of the genes observed to be repressed by blue light that are dependent on either cry1 (41/138 = 30%) or HFR1 (41/263 = 16%) (Figure 2B). This suggests that HFR1 is more likely a turn-on switch to light-induced genes rather than a shutdown of genes expressed in the dark.

In the 293 genes that were significantly induced by blue light and dependent on both cry1 and HFR1, 50% (146 of 293) were induced by more than 10 fold (Data S2, S3, S4, S5). The 293 genes induced by cry1 (WT/*cry1*) in blue light were also very robustly induced by blue light in WT compared to those in the dark (B/D) (Figure 2C). When these 293 genes were ranked by blue light induction in WT, there was no blue light response observed for these 293 genes in *cry1* mutants, and a significantly reduced blue light response was found for these 293 genes in *hfr1* mutants; suggesting that cry1 is required for blue light induced gene expression and that HFR1 is required to elevate their expression levels. A few genes involved in electron transport were induced by more than 100 fold by 1 h of blue light and dependent on both cry1 and HFR1 (Data S2); suggesting that there was a highly active electron flux after the seedlings were exposed to 1 h of blue light. The majority of the 293 genes were induced by less than 100 fold (Data S3, S4, S5). These gene products are involved in a large number of bioprocesses (Figure 2D); indicating that blue light potentially stimulates the entire system to initiate photomorphogenesis through both cry1 and HFR1. There are also some genes regulated by blue light that are dependent on either cry1 or HFR1 in the same comparison; indicating that a small portion of the blue light signal perceived by cry1 is processed by factors other than HFR1 to regulate downstream gene expression. Meanwhile, HFR1 can also process blue light signals perceived by photoreceptors other than cry1.

**Figure 3:**
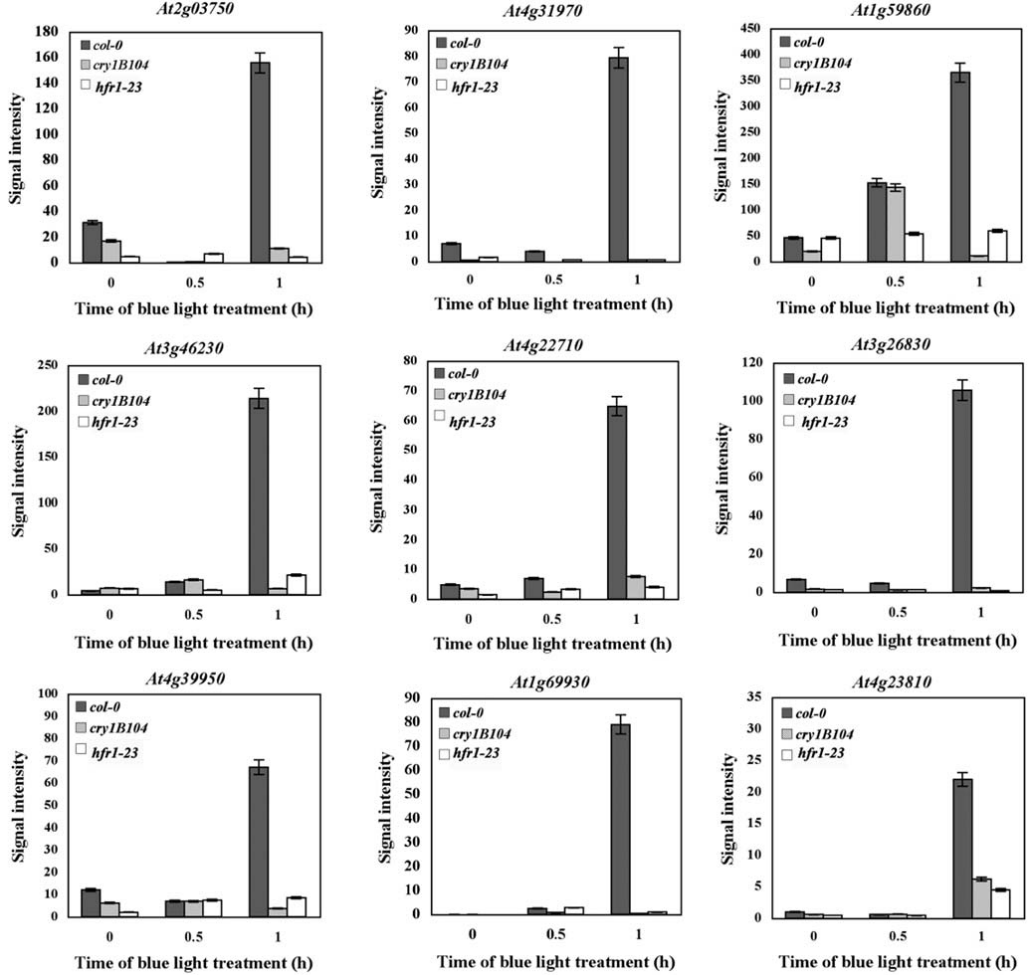
Dynamic change of gene expression from dark, 30 min and 1 h blue light. RT-PCR of selected genes from 4-day-old dark-grown WT, *cry1* and *hfr1* mutant seedlings treated by dark, 30 min and 1 h blue light (50 *µmol m^−2^ s^−1^*). Relative expression level of each gene was normalized to *GAPDH*. Expression level was from the average of three biological replicates.

To examine the dynamic changes in gene expression in response to early blue light, selected genes were tested for their expression patterns in the dark, and after treatment by 30 min and 1 h of blue light (Figure 3). In the first 30 min of blue light treatment, there was only a moderate change in expression in WT and in either mutant. The expression levels of these genes was dramatically increased after 1 h in the WT, while this drastic change was not found either in dark grown plants or in either mutant. This significant blue light induction seemed to happen only in WT seedlings treated with blue light from 30 min to 1 h.

Notably, a number of cytochrome P450 genes and stress/defense-related genes were induced by blue light and dependent on both cry1 and HFR1. To see if these genes were regulated by common *cis*-elements, all of the known plant promoter motifs from www.arabidopsis.org were used to generate a plant motif database. All upstream 500 bp sequences from the *Arabidopsis* genome were used as background control. By using PRIMA, the overrepresented motifs were selected. In blue light-induced genes that were dependent on both cry1 and HFR1, two motifs, a W-box and an OCS element, were found highly overrepresented with p-values even lower than 10^−10^(Table 1). W-boxes were found in most (78%) of the promoters and OCS elements were found in 21% of the promoters, indicating that HFR1 may recognize genes containing these *cis*-elements regardless of their biological functions.

**Table 1:** Motifs overrepresented in genes induced by blue light and dependent on both cry1 and HFR1.

MOTIFS	SEQUENCES	P VALUE
W-box	**TTGAC**	5.30E-11
OCS	TG**ACG**(C/**T**)AAG(C/G)(A/G)(A/C)T(G/T)**ACG**(C/**T**)(A/C)	4.20E-10
***CYP82C2***		
ACAAAAACAAAAAAATATAATTAGTGTATTTTAAATTATTTGTGTTTTAATATATTTTTTATTTTATCTTTTAAAACATGTTTTTTATTTGAGTTATTATTATGAATTCAGTATTATAAAGTCATATCTTCATTTCAATTTTTTTTTGAATATAATGTTATATAATATTTCTAAACACAAGTAGATAACGTTG***GTCAA***TATTTGGTTAAGATAAATGGTGGAAAAATATTCAGAAATGTTCAAAAATGTTCGACCATTTTTTTTATTTCAAAATGTACGTCAGTAACTATCGATTTTTT***TTGAC***CATATACAATTTGCGACCCCCGCCTTTCGACGACTTGCTTTTG***GTCAA***ACAGCAGTAAGATAGGCGTATGTCTCATGCTTACATGGTATTGAACCGATAATATGTGTGTGTATATATAGAGAGACAGACTATACTTTTTAATCATTCAAAACTAGAAATCACCAAACACACATCTCTTTTGCACGCTCAAACCACT		

The consensus sequences were emphasized by *bold*. Examples of W-box sequences were highlighted by *bold* and *italics* in 500 bp upstream sequences in *CYP82C2*.

### Both HFR1 and cry1 are crucial for *CYP82C2* promoter activity in blue light

**Figure 4:**
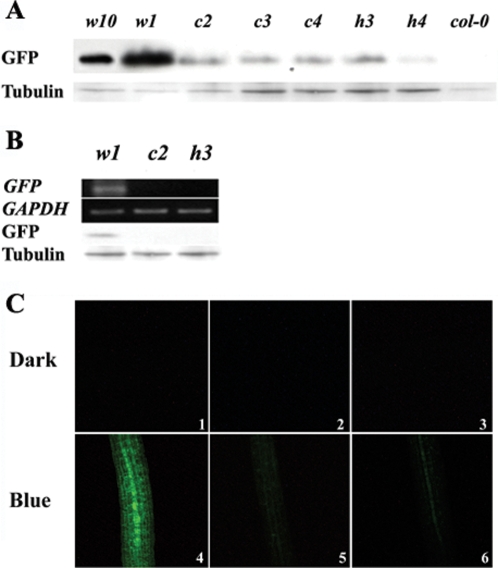
Activity of the *CYP82C2* promoter depends on both cry1 and HFR1 and requires blue light. (A) GFP protein level in independent *P* *_CYP82C2_* *::GFP* transgenic lines grown in blue light (50 *µmol m^−2^ s^−1^*) for 7 days. WT was used as a negative control for GFP detection; *w1* and *w10*: independent transgenic lines in *col-0* background; *c2*, *c3* and *c4*: independent transgenic lines in *cry1* background; *h3* and *h4*: independent transgenic lines in *hfr1* background. Tubulin was used as loading control. (B) *GFP* expression on RNA and protein level in *w1*, *c2* and *h3* transgenic plants after 1 h blue light (50 *µmol m^−2^ s^−1^*) treatment. *GAPDH* was used as the control for RT-PCR; tubulin was used as control in western blot. (C) GFP fluorescence in 7-day-old transgenic seedlings grown in darkness and continuous blue light (50 *µmol m^−2^ s^−1^*). 1 and 4: *P_CYP82C2_::GFP/col-0*; 2 and 5: *P_ CYP82C2_::GFP/cry1*; 3 and 6: *P_ CYP82C2_::GFP/hfr1*.

The cytochrome P450 gene, *CYP82C2*, was identified as one of the genes extremely responsive to early blue light and dependent on both cry1 and HFR1. Microarray analysis (Data S2) and PCR results (Figure 3) showed that its expression was strongly influenced by the presence/absence of both cry1 and HFR1 in blue light. Three W-box motifs were found in its 500 bp upstream region (Table 1). Therefore, we cloned the basic promoter region from *CYP82C2* and generated transgenic plants carrying the *CYP82C2* promoter driving *GFP* (*Pcyp_82C2_::GFP*) in WT, *cry1* and *hfr1* backgrounds to examine the promoter activity influenced by light treatment and cry1/HFR1. Transformants screened by hygromycin B resistance were self-fertilized, and the homozygous progeny were selected in the T4 generation. Multiple independent lines were generated and studied in each genetic background (Figure 4A). GFP protein was found highly expressed in both *Pcyp_82C2_::GFP/col-0* transgenic lines, while only weak protein signal was detected in all the *Pcyp_82C2_::GFP*/*cry1* and *Pcyp_82C2_::GFP*/*hfr1* lines. This different GFP expression level between WT and mutants was consistent in multiple transgenic lines; supporting that it reflected the actual effect of cry1 and HFR1, but was not due to the insertion difference. After a 1 h blue light treatment, *GFP* expression was detected only in transgenic plants in WT background on both RNA and protein levels, but not in transgenic plants that were in either *cry1* or *hfr1* backgrounds (Figure 4B). GFP fluorescence was barely seen in any of the transgenic seedlings grown in the dark. After the seedlings were grown in blue light for 7 days, the fluorescence signal became very strong in *Pcyp_82C2_::GFP/col-0* seedlings, but a much weaker signal was detected in either *Pcyp_82C2_::GFP*/*cry1* and *Pcyp_82C2_::GFP*/*hfr1* seedlings (Figure 4C). GFP fluorescence was detectable in every tissue, with the strongest signal in leaves and hypocotyls in WT, while it was very faint everywhere in *cry1* and *hfr1* mutants. No tissue specific expression difference was observed among all the transgenic seedlings. This suggests that the presence of cry1 and HFR1 are crucial for the *CYP82C2* promoter activity and that blue light is required for this event to occur.

### Light regulated *HFR1* RNA and protein dynamics in *cry1* mutants and wild-type seedlings

**Figure 5:**
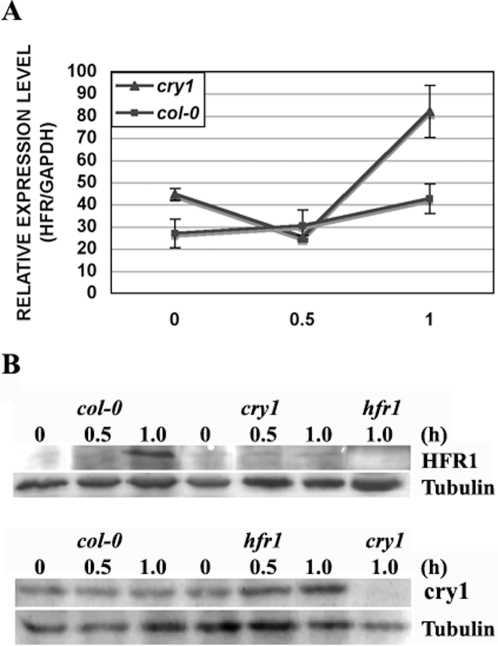
cry1 influences HFR1 on both RNA and protein level. (A) RT-PCR showing the relative expression level of *HFR1* normalized to *GAPDH*. Expression levels were the average of three biological replicates. (B) Western blot showing cry1 and HFR1 protein level in WT, *cry1* and *hfr1* mutants in the dark, 30 min and 1 h blue light (50 *µmol m^−2^ s^−1^*). Tubulin was used as the control.

To gain more knowledge about the difference between WT and *cry1* mutants, we examined *HFR1* RNA and protein levels in the dark, and after 30 min and 1 h of blue light treatment. *HFR1* RNA was found to be linearly increased from dark, to 30 min and 1 h of blue light in WT, while the levels were more than 2 fold greater in *cry1* mutants at 1 h of blue light than in WT (Figure 5A). This suggested that the absence of cry1 induced *HFR1* RNA synthesis or stability, which is in agreement with what the Fankhauser group had observed earlier [6]. In contrast to the RNA level, HFR1 protein accumulation was barely detectable in the dark and was significantly increased between 30 min and 1 h of blue light treatment in WT. However, this pattern disappeared in *cry1* mutants. Only a weak band was detected in *cry1*, while it was undetectable in *hfr1* mutants; indicating that HFR1 protein accumulation depends on the presence of cry1. However, cry1 protein was constantly present at comparable levels in either WT or *hfr1* mutants (Figure 5B). Note that the cry1 protein level is not correlated with the global gene expression changes between the two mutants, while HFR1 protein abundance is indeed correlated with the expression profiles in the early stages (1 hour) of photomorphogenesis that are induced by blue light (Figure 2A). Combining both protein and RNA levels of HFR1 that we detected, it is possible that the accumulation of HFR1 protein itself provides a negative feedback signal to its transcription, and this feedback signal is attenuated in *cry1* mutants due to a significantly reduced amount of HFR1 protein.

## Discussion

From skotomorphogenesis to photomorphogenesis, plants initiate a dark/light transition, in which related genes are differentially expressed. It has been shown that the expression profiles under different light qualities are similar to each other [21]; indicating that photomorphogenesis, mediated by the primary photoreceptors in different light conditions (blue, red and far-red), is triggered by similar transcriptional machinery. It is possible that common factors join in integrating different signals into global transcriptional regulation [22]. Genes that keep plants etiolated have to be turned off. To start de-etiolation, light responsive genes, especially those crucial to development, need to be turned on, so that plants are able to survive and respond to light for further growth and development. Therefore, the factors involved in this regulatory machinery are crucial for later events.

Several independent microarray studies have been carried out to investigate global gene expression in response to blue light [23]–[26]. Most of them were focused on much later seedling stages than a 1 h treatment. The Spalding group [23] has looked at cry1-responsive gene profiles at a much higher blue light irradiance (100 *µmol m^−2^ s^−1^*) after a shorter time (45 min). They found that approximately 5% of the genes presented on the 8K Genome Arrays were differentially expressed in *cry1* mutants under their conditions. These genes were those that encode products that are involved in different bioprocesses. We used a much lower blue light irradiance (50 *µmol m^−2^ s^−1^*) to avoid high irradiance-caused damage to plant cells. In addition, the *Affymetrix* ATH1 22K Arrays have greater capability to look at the entire genome, with many more genes that are missing on the 8K arrays. Considering the high sensitivity of the microarray approach, and various experimental conditions and arrays that were used, it is not surprising that all these microarray profiles, including ours, are not comparable to each other. In addition to the interest in cry1, we also looked at HFR1 which has a role in the blue light signaling pathway has not been elucidated at the genomic level before. The most interesting result from the 2-way ANOVA analysis was a list of genes changed due to the interaction of blue light and genotype. A significant interaction, quantified by p-value, means the effect of one variable (blue light) changes depending on the level of the other factor (cry1 and HFR1), which provides insight into the biological pathways induced by blue light and activated by cry1 and HFR1, rather than the simple cumulative effect of blue light and cry1/HFR1. It is notable that genes with low p-values for their genotype and light treatment may, or may not, be due to the interaction of both factors. Given the knowledge that cry1 is the predominant, but not the only blue light receptor, it is possible that some blue light responsive genes are able to have a low p-value for cry1 and blue light separately, but that these two factors are independent of each other. Other blue light receptors might be involved in light perception by also triggering gene expression changes. Therefore, these blue light regulated gene changes are not specifically cry1-mediated. In light of our interest in gene expression, specifically regulated by blue light through both cry1 and HFR1, introducing the concept of the interaction between genotype and blue light treatment can rule out non-specificity and provide a way to look at gene profiling without the interference from other blue light photoreceptors.

Our data suggest that HFR1 may be responsible for the majority of transcriptional regulation in cry1 signaling. In an earlier study, cryptochromes were shown to be the major blue light receptors for gene expression regulation [25]. To-date, three cryptochromes (cry1, cry2 and cry-DASH) have been found in plants. Both cry2 and cry-DASH were shown to have DNA-binding ability and cry2 was localized in nucleus, while cry-DASH was found in chloroplasts and mitochondria [27]–[29]. Phytochrome A and phototropins have been recognized as blue light receptors as well. In addition, several downstream regulators were also found to positively or negatively regulate blue light signaling, such as HFR1, HY5, HRB1, PP7, RPT2, NPH3, OBP3, SHB1 *etc*. [5], [6], [9]–[12]. The blue light signaling pathway may involve all or some of these regulators in the entire system to control global gene expression. In our case, cry1 and HFR1 significantly induced a large number of common gene targets, while they showed little regulatory function at the transcriptional level in the dark. Similarly, blue light signaling was similarly repressed when either cry1 or HFR1 was missing. This suggests that HFR1 may play a key role in regulating gene expression in cry1 signaling in response to blue light. Several cytochrome P450 genes (*CYP71A12*, *CYP71B15*, *CYP82C2* and *CYP81F2*, *etc.*) were strikingly induced, while all of their expression in the dark or in mutants was quite low. A similar expression pattern was also observed in the rest of the 293 genes (Figure 2C, Data S2, S3, S4, S5). None of these P450 genes have been reported in early blue light responses before [22], [24], [25], [30]. An emphasis on the flavin-binding nature of cry1 shows that the intracellular redox state of the flavin is the key to the regulation of cry1 activity in blue light perception [31], [32]. Blue light excites FAD to accept electrons from intrinsic donors (Trp and Tyr) [32]. The highly induced P450 genes may be the products of the redox homeostasis adjustment that is triggered by the new electron flux related to cry1 function. The other products of redox flux are reactive oxygen species [33], which generates an interface for many biotic/abiotic stresses, such as pathogen infection, water, and temperature stresses, and physiological reactions, such as those involved in hormone and defense responses [33]. Our data provide insight into the blue light regulation of these genes that depends on the presence of cry1/HFR1.

It has been reported that cry1 can interact with COP1, an E3 ligase that is involved in HFR1 degradation [17], [18], [34], [35]. We found that only a trace amount of HFR1 protein was detected in *cry1* mutants after 1 h in blue light, which makes *cry1* mutants function as an *hfr1* “knockdown” allele. It is likely that the similar level of HFR1, but not cry1, between *cry1* and *hfr1* is responsible for global gene expression profiles. There is a correlation between the expression changes in selective genes from the dark, 30 min to 1 h blue light treatment and the dynamic HFR1 protein level with the strongest HFR1 protein accumulation detected at 1 h of blue light (Figure 5B). As previously suggested by a high-resolution kinetic study of blue light-mediated hypocotyl growth inhibition, the hypocotyl elongation rate is indistinguishable in darkness and during the first 30 min in blue light between WT and *cry1* mutants, while it becomes significantly distinguishable after 30 min. It has been suggested that cry1 takes over control of photomorphogenesis from phototropins only after 30 min of blue light irradiation [36]. Our data suggest that the cry1-mediated blue light induced transcriptional machinery is likely evoked no earlier than 30 min after blue light exposure, and HFR1 seems to be the key to this machinery. The presence of cry1 negatively correlates with *HFR1* RNA levels (Figure 5A). Combining both protein and RNA level of HFR1 that we detected, it is possible that the accumulation of HFR1 protein itself provides a negative feedback signal to its transcription, and this feedback signal is attenuated in *cry1* mutants due to a significantly reduced amount of HFR1 protein.

Dissection of transcriptional networks is one of the greatest challenges of functional genomics. Usually, genes that are co-expressed over multiple biological conditions may share common regulatory elements in their promoters. Our data show that genes that were induced by blue light and dependent on both cry1 and HFR1 encode products with very diverse biological functions. The repression of their expression in *cry1* and *hfr1* mutants may cause pleoitropic phenotypes in the mutants. In a previous study, a G-box (CACGTG) element was found enriched in high irradiance-responsive genes that were regulated by cry1 and HY5 [37]. Phytochrome-interacting factors bind to G-boxes in red/far red light pathways as well [38]. Instead of G-boxes, we found that W-boxes and OCS elements were significantly overrepresented in the upstream 500 bp region in the blue light-induced genes that were dependent on both cry1 and HFR1. The W-box is a WRKY binding motif, which is the specific binding site of a transcription factor family related to senescence and defense systems in plants. W-boxes were found in most of the induced genes, such as the cytochrome P450 genes *CYP82C2*, *CYP81F2* and *CYP71A12*. The W-box-containing promoter in *CYP82C2* gene is fully functional only when both cry1 and HFR1 are present (Figure 4B and C). The OCS element is a consensus sequence found in plant promoters that have been strongly linked to plant defense and stress response genes [39], [40], such as GST and heat shock protein genes. It seems that a series of stress responsive/defense-related mechanisms are turned on as photomorphogenesis starts. Such a significant overrepresentation of these two motifs is likely due to a direct binding of specific transcription factors that are activated by the signal from cry1 through HFR1. It is also possible that HFR1 induces the expression of the transcription factors that bind to these motifs to elevate the expression of the gene targets. It will be helpful to further dissect the role that HFR1 plays in gene regulation when such a promoter analysis is done in other light conditions.

In conclusion, our study on cry1 and HFR1 specific global gene expression profiles provides an intriguing way to analyze the transcriptional machinery evoked by blue light as well as a genome-wide profile regulated by HFR1. For the first time, *cry1* and *hfr1* have shown striking similarities in the “microarray phenotype” during blue light treatment. This phenotype is correlated with HFR1 protein abundance indicating that HFR1 plays a key role in sorting out the cry1-mediated blue light signal and sending it to the entire genome. On the other hand, the presence of cry1 is required for HFR1 protein accumulation in blue light. Without HFR1, even when cry1 is present, as in the case in *hfr1* mutants, the signal sent by cry1 cannot be properly delivered. We also propose that the cry1-mediated blue light signal is possibly delivered to W-box and OCS element-containing genes, although it is still unclear whether they are direct targets of HFR1, or whether other W-box and OCS element binding factors are targeted by HFR1. It will also help to reveal the larger picture of the cry1 signaling pathway by examining the protein dynamics of other factors in the blue light pathway in *cry1* mutants, in addition to cry1 itself.

## Materials and Methods

### Plant Growth Conditions and Mutant Screening

All *Arabidopsis thaliana* plants used here are from the Columbia (*Col*) background. T-DNA pools (CS31087) for mutant screening are from the *Arabidopsis Biological Resource Center*. Light sources were as described previously [41], except for far-red light which was provided by LEDs (λmax at 739 nm). For most experiments, seeds were surface sterilized, plated on MS medium containing 3% Sucrose and 0.8% agar and stratified in the dark at 4°C for 4 days before monochromatic light treatment at 22°C.

Blue light (8 *µmol m^−2^ s^−1^*) was applied for blue light insensitive mutant screening. After 4 days of blue light treatment, seedlings showing long hypocotyl, or unopened cotyledon phenotypes were transferred into soil for seed setting. In the next generation, all the mutant candidates were retested in blue, red, far-red and dark conditions. They were then backcrossed three times with WT plants to increases isogenicity.

For the microarray study, seedlings were grown in the dark for 4 days followed by a 1 h blue light (50 *µmol m^−2^ s^−1^*) treatment. The dark treated seedlings were kept in the dark before harvest.

About 400 ng of genomic DNA, isolated from *hfr1-23* plants, was digested by *Dra* I, *Eco*R V, *Pvu* II, *Sca* I, and *Ssp* I restriction enzymes and linked to an adapter duplex to produce five genomic-walk libraries. T-DNA-flanking genomic regions were amplified using a pair of nested primers specific to the T-DNA borders in combination with a pair of nested primers (AP1, AP2) specific to the adapters of the Clontech GenomeWalker kit. Genomic regions flanking the T-DNA insertions in the *hfr1-23* mutants were isolated by PCR [42]. All primer sequences are shown in Data S7.

### Probe Preparation and Hybridization

To prepare “probe” RNA for microarray hybridization, 5 µg of total RNA was primed with 100 pmol oligo dT (24) primer containing a 5′ T7 RNA polymerase promoter sequence. First strand cDNA was synthesized using 200 units of SuperScript™ II RNase H^−^ Reverse Transcriptase (Invitrogen) at 42°C for 2 hr. Second strand synthesis was performed using a cocktail (10 units *E. coli* DNA ligase, 40 units DNA polymerase I, 2 units RNase H), with the second strand buffer (invitrogen) and incubated at 16°C for 2 hr, followed by another 5 min incubation at 16°C after adding 10 units T4 DNA polymerase to each reaction. The reaction was then terminated by 10 µL of 0.5 M EDTA. The (ds)-cDNA product was further purified using an equal volume of phenol: chloroform: isoamyl alcohol (25∶24∶1, saturated with 10 mM Tris-HCl pH 8.0/1 mM EDTA) on a phase lock gel and then precipitated by adding 0.5 volume of RNase free 7.5 M NH_4_Ac and 2.5 volumes of −20°C RNase free 100% ethanol. The purified cDNA was used as a template for generating biotinylated cRNA probes via BioArray™ High Yield™ RNA transcription labeling kit (T7) (Enzo) at 37°C for 5 hrs. The biotin labeled cRNA was purified by Qiagen RNeasy spin columns then fragmented at 94°C for 35 min to produce cRNA oligomers about 50–200 bp long.

Affymetrix (Santa Clara, CA, USA) GeneChip Arabidopsis ATH1 Genome Arrays were used to detect differences in gene expression. Twenty micrograms of fragmented cRNA was used for hybridization. Hybridization and scanning was done by the microarray facility in the University of Pennsylvania.

### Statistical Analysis

All genes from three biological replicates of either WT or mutants were evaluated by GeneSpring v7.2 (Silicon Genetics). The .CEL files from microarray data were input into GeneSpring in GCRMA format. Expression values for all probe sets were used to generate Principle Component Analysis (PCA) plots.

The expression values for all probe sets were then input into Partek software for statistical analysis. All samples were defined by two parameters: genetic background and light treatment. A 2-way ANOVA analysis was performed in the comparison of WT/*cry1* and WT/*hfr1* using all of the probes. A p-value of each probe was calculated for statistical significance. All genes were evaluated and put into three different categories: genotype (G), light treatment (L) and the interaction of genotype and light treatment (G*L). The p-value cutoff for each category was calculated according to the false discovery rate at a significance level of 0.001. All probes with p-values lower than the false discovery rate of 0.001 for G*L were selected to calculate the mean fold changes in blue light within each comparison. Genes having more than 2 fold changes (including 2 fold) with p-values for interactions of G*L lower than false discovery rate of 0.001 significance were selected as genes that were significantly and robustly regulated by blue light, depending on their genetic background.

### RT-PCR Analysis

RNeasy Mini Kit was used to isolate total RNA. Five micrograms of total RNA from each sample was applied for reverse transcription. The transcription level of selected candidate genes was verified by RT-PCR using *GAPDH* as the control. The cDNA templates and PCR cycles were optimized for linear amplification. Resulting signals were photographed and quantified by Kodak 1D™ Gel Analysis software 3.6.3. All primers are listed in Data S7.

### Promoter Analysis

The 500-bp upstream sequences of each gene from different gene lists (induced/repressed by blue light that are dependent on both cry1 and HFR1) were extracted from a FASTA file of promoter sequences downloaded from www.arabidopsis.org. These promoter sequences, and the plant motif database generated from the above website, were used as input to a program called Promoter Integration in Microarray Analysis (PRIMA v1.0) [43]. The overrepresented motifs in each gene list were selected by P value lower than 10^−5^. All upstream 500 bp sequences from the whole genome were used as the background control.

### Immunoblot Analysis

Seedlings were harvested and ground in liquid nitrogen for protein preparation. The crude proteins were extracted using the CelLytic™ P plant cell lysis/extraction reagent (Sigma) with a 1∶100 dilution of a protease inhibitor cocktail (Sigma). All the protein samples were loaded onto 10% SDS-PAGE gels for separation. The proteins were then transferred to Hybond™-ECL™ nitrocellulose membranes (Amersham Biosciences), blocked with a 1% blocking reagent (Roche) in PBST and probed with cry1, HFR1 antisera (generated from full-length cDNA by Covance Inc.) and β-Tubulin antibody (Sigma). An ECL system was used for final detection.

### Analysis of CYP82C2 promoter efficiency in different genetic background

An 800 bp of *CYP82C2* promoter fragment upstream of ATG was cloned by PCR from WT genomic DNA. *Kpn* I and *Nco* I sites were generated for further cloning by two primers; P82C2FKPNI: 5′-CCG GTA CCA GTG GAA TGG CGA GGC AAA T-3′, and P82C2RNCOI: 5′-GGC CAT GGC ATT CAT CCA TAG TGG TTT GAG CGT GCA AAA GAG ATG TGT G-3′. Then the promoter fragment was cloned into *Kpn* I/ *Nco* I sites on pCAMBIA 1302 by substitution of 35S promoter in the front of *GFP* gene. Then the construct was transferred to *Agrobacterium tumefaciens* C58 to generate transgenic lines in WT, *cry1* and *hfr1* background. After screening and selection, the T4 generation was planted on MS plates and grown in dark for 4 days followed by 1 h blue light treatment or constant blue light for 7 days (50 *µmol m^−2^ s^−1^*). Five seedlings were picked from each line for protein detection. GFP fluorescence in each line was observed and photographed by Leica Firecam.

ArrayExpress accession: E-MEXP-1784

## Supporting Information

Data S1 Gene expression profiles of all 22,810 genes on the Arabidopsis Affymetrix ATH1 microarray.
(8.57 MB XLS)

Data S2 Genes induced by blue light in cry1- and HFR1-dependent manner with MFI> = 100.
(0.08 MB DOC)

Data S3 Genes induced by blue light in cry1- and HFR1-dependent manner with 100>MFI> = 10.
(0.27 MB DOC)

Data S4 Genes induced by blue light in cry1- and HFR1-dependent manner with 10>MFI> = 5.
(0.19 MB DOC)

Data S5 Genes induced by blue light in cry1- and HFR1-dependent manner with 5>MFI> = 2.
(0.15 MB DOC)

Data S6 Genes repressed by blue light in cry1- and HFR1-dependent manner.
(0.12 MB DOC)

Data S7 Primers for PCR amplification
(0.02 MB DOC)
