# Supplementary material for: HFR1 Is Crucial for Transcriptome Regulation in the Cryptochrome 1-Mediated Early Response to Blue Light in Arabidopsis thaliana
Source: PLoS One. 2008 Oct 30;3(10):e3563. doi: 10.1371/journal.pone.0003563 (PMC2570330; doi:10.1371/journal.pone.0003563)
Supplement: Data S2 — Genes induced by blue light in cry1- and HFR1-dependent manner with MFI> = 100. (0.08 MB DOC) [file pone.0003563.s002.doc]

**Data S2 Genes induced by blue light in cry1- and HFR1-dependent manner with MFI≥100.**

| **AGI Locus** | **Gene Description** | **^a^MFIB(w/c)** | **^b^MFI_*WT*(B/D)** | **^c^MFI_*cry1*(B/D)** | **^d^MFI_*hfr1*(B/D)** |
| --- | --- | --- | --- | --- | --- |
| **Electron transport** | |  |  |  |  |
| AT1G26380 | FAD-binding domain-containing protein | 514.38 | 121.03 | 0.37 | 11.71 |
| AT2G30750 | cytochrome P450 71A12, putative (CYP71A12) | 513.49 | 144.47 | 0.49 | 16.85 |
| AT3G26830 | cytochrome P450 71B15, putative (CYP71B15) | 233.04 | 100.10 | 0.47 | 6.05 |
| AT4G31970 | cytochrome P450 family protein | 231.60 | 130.00 | 1.19 | 1.86 |
| AT5G57220 | cytochrome P450, putative | 226.50 | 61.29 | 0.24 | 3.26 |
| **Metabolism** |  |  |  |  |  |
| AT2G26560 | patatin, putative | 326.46 | 104.13 | 0.45 | 6.74 |
| AT2G28210 | carbonic anhydrase family protein | 299.78 | 157.09 | 1.16 | 4.88 |
| AT1G69930 | glutathione S-transferase, putative | 161.81 | 109.23 | 0.97 | 2.63 |
| AT3G60140 | glycosyl hydrolase family 1 protein | 154.89 | 55.88 | 0.99 | 4.27 |
| AT1G67980 | caffeoyl-CoA 3-O-methyltransferase, putative | 137.97 | 88.37 | 1.03 | 1.34 |
| **Stress-induced/defense, senescence-related** | |  |  |  |  |
| AT3G46230 | 17.4 kDa class I heat shock protein (HSP17.4-CI) | 286.84 | 81.73 | 0.77 | 1.55 |
| AT5G12030 | 17.7 kDa class II heat shock protein 17.6A (HSP17.7-CII) | 248.34 | 60.26 | 0.58 | 1.92 |
| AT2G41260 | glycine-rich protein / late embryogenesis abundant protein (M17) | 188.55 | 19.56 | 0.64 | 0.22 |
| AT1G59860 | 17.6 kDa class I heat shock protein (HSP17.6A-CI) | 155.85 | 176.05 | 1.36 | 4.35 |
| AT1G53540 | 17.6 kDa class I small heat shock protein (HSP17.6C-CI) (AA 1-156) | 139.22 | 94.19 | 0.91 | 1.74 |

a: MFIB (w/c): Mean fold induction in gene expression between *WT* and *cry1* in blue light;

b: MFI_*WT* (B/D): Mean fold induction in gene expression between blue light and the dark in *WT*;

c: MFI_*cry1* (B/D): Mean fold induction in gene expression between blue light and the dark in *cry1* mutants;

d: MFI_*hfr1* (B/D): Mean fold induction in gene expression between blue light and the dark in *hfr1* mutants.
