# Supplementary material for: HFR1 Is Crucial for Transcriptome Regulation in the Cryptochrome 1-Mediated Early Response to Blue Light in Arabidopsis thaliana
Source: PLoS One. 2008 Oct 30;3(10):e3563. doi: 10.1371/journal.pone.0003563 (PMC2570330; doi:10.1371/journal.pone.0003563)
Supplement: Data S3 — Genes induced by blue light in cry1- and HFR1-dependent manner with 100>MFI> = 10. (0.27 MB DOC) [file pone.0003563.s003.doc]

**Data S3 Genes induced by blue light in cry1- and HFR1-dependent manner with 100>MFI ≥10.**

| **AGI Locus** | **Gene Description** | **^a^MFIB(w/c)** | **^b^MFI_*WT*(B/D)** | **^c^MFI_*cry1*(B/D)** | **^d^MFI_*hfr1*(B/D)** |
| --- | --- | --- | --- | --- | --- |
| **Electron transport** |  |  |  |  |  |
| AT3G14620 | cytochrome P450, putative (CYP72A8) | 42.77 | 62.46 | 1.77 | 2.22 |
| AT1G26390 | FAD-binding domain-containing protein | 41.33 | 49.61 | 1.07 | 2.18 |
| AT4G37370 | cytochrome P450, putative (CYP81D8) | 36.95 | 15.36 | 0.36 | 1.29 |
| AT4G39950 | cytochrome P450, putative (CYP79B2) | 33.11 | 17.15 | 0.49 | 1.53 |
| AT1G26410 | FAD-binding domain-containing protein | 27.18 | 28.23 | 1.03 | 1.17 |
| AT1G26420 | FAD-binding domain-containing protein | 19.93 | 17.11 | 1.02 | 1.71 |
| AT4G22710 | cytochrome P450 family protein (CYP706A2) | 18.87 | 10.06 | 0.77 | 0.42 |
| AT1G30700 | FAD-binding domain-containing protein | 18.66 | 16.91 | 1.40 | 3.67 |
| AT5G20230 | plastocyanin-like domain-containing protein | 17.94 | 7.40 | 0.26 | 0.76 |
| AT2G30770 | cytochrome P450 71A13, putative (CYP71A13) | 16.65 | 14.73 | 0.89 | 1.18 |
| AT1G28480 | glutaredoxin family protein | 13.39 | 6.53 | 0.49 | 1.35 |
| AT2G24180 | cytochrome P450 family protein (CYP71B6) | 12.21 | 7.27 | 0.64 | 1.16 |
| **Growth and development related proteins** | |  |  |  |  |
| AT3G22640 | cupin family protein | 67.81 | 8.06 | 0.54 | 0.62 |
| AT4G28520 | 12S seed storage protein, putative / cruciferin, putative | 47.07 | 12.14 | 0.82 | 1.23 |
| AT5G44120 | 12S seed storage protein (CRA1) | 35.30 | 8.14 | 0.41 | 1.16 |
| AT2G28490 | cupin family protein | 23.43 | 9.53 | 1.01 | 1.00 |
| AT2G46240 | IQ domain-containing protein / BAG domain-containing protein | 23.38 | 18.04 | 1.16 | 0.90 |
| AT1G26240 | proline-rich extensin-like family protein | 22.15 | 24.76 | 1.10 | 1.22 |
| AT1G22890 | expressed protein | 13.88 | 13.57 | 0.91 | 1.19 |
| **Hormone-related** |  |  |  |  |  |
| AT4G09600 | gibberellin-regulated protein 3 (GASA3) | 16.30 | 13.89 | 1.20 | 1.01 |
| **Metabolism** |  |  |  |  |  |
| AT1G69920 | glutathione S-transferase, putative | 61.05 | 43.62 | 0.33 | 5.11 |
| AT1G54870 | short-chain dehydrogenase/reductase (SDR) family protein | 56.65 | 23.44 | 0.82 | 1.12 |
| AT1G74590 | glutathione S-transferase, putative | 49.52 | 21.74 | 0.57 | 1.13 |
| AT3G60120 | glycosyl hydrolase family 1 protein | 48.03 | 43.64 | 0.91 | 1.14 |
| AT3G49620 | 2-oxoacid-dependent oxidase, putative (DIN11) | 47.20 | 43.53 | 1.15 | 1.41 |
| AT5G22300 | nitrilase 4 (NIT4) | 43.47 | 48.96 | 1.51 | 3.72 |
| AT5G38900 | DSBA oxidoreductase family protein | 39.53 | 25.17 | 0.62 | 1.59 |
| AT3G05260 | short-chain dehydrogenase/reductase (SDR) family protein | 36.19 | 20.94 | 0.99 | 0.91 |
| AT3G21370 | glycosyl hydrolase family 1 protein | 29.22 | 8.09 | 0.96 | 0.95 |
| AT5G02780 | In2-1 protein, putative | 27.88 | 18.50 | 1.01 | 3.10 |
| AT1G23730 | carbonic anhydrase, putative / carbonate dehydratase, putative | 27.57 | 14.24 | 1.09 | 2.75 |
| AT5G40990 | GDSL-motif lipase/hydrolase family protein | 24.94 | 26.13 | 1.05 | 1.19 |
| AT1G22400 | UDP-glucoronosyl/UDP-glucosyl transferase family protein | 21.21 | 12.43 | 0.48 | 0.92 |
| AT5G48570 | peptidyl-prolyl cis-trans isomerase, putative | 20.63 | 20.92 | 1.33 | 1.09 |
| AT1G05680 | UDP-glucoronosyl/UDP-glucosyl transferase family protein | 20.62 | 3.76 | 0.19 | 0.73 |
| AT1G09500 | cinnamyl-alcohol dehydrogenase family | 17.59 | 18.51 | 1.06 | 1.10 |
| AT2G39030 | GCN5-related N-acetyltransferase (GNAT) family protein | 17.15 | 17.31 | 1.04 | 1.03 |
| AT1G17170 | glutathione S-transferase, putative | 16.59 | 6.21 | 0.30 | 0.84 |
| AT3G02800 | tyrosine specific protein phosphatase family protein | 13.85 | 9.86 | 0.65 | 1.06 |
| AT2G15480 | UDP-glucoronosyl/UDP-glucosyl transferase family protein | 13.39 | 6.19 | 0.48 | 0.84 |
| AT1G30370 | lipase class 3 family protein | 13.28 | 6.67 | 0.67 | 0.95 |
| AT1G21120 | O-methyltransferase, putative | 13.27 | 9.72 | 0.90 | 2.15 |
| AT1G18590 | sulfotransferase family protein | 12.84 | 7.50 | 0.81 | 1.17 |
| AT2G29460 | glutathione S-transferase, putative | 12.79 | 13.62 | 1.40 | 1.80 |
| AT2G15490 | UDP-glucoronosyl/UDP-glucosyl transferase family protein | 12.71 | 4.92 | 0.30 | 0.82 |
| AT1G74460 | GDSL-motif lipase/hydrolase family protein | 12.41 | 5.98 | 0.66 | 1.27 |
| AT1G32940 | subtilase family protein | 10.87 | 11.89 | 1.08 | 1.65 |
| AT1G76690 | 12-oxophytodienoate reductase (OPR2) | 10.05 | 9.04 | 1.10 | 2.10 |
| **Photosysthesis/chloroplast proteins** | |  |  |  |  |
| AT2G29500 | 17.6 kDa class I small heat shock protein (HSP17.6B-CI) | 41.54 | 19.44 | 0.90 | 1.73 |
| AT5G05730 | anthranilate synthase, alpha subunit | 31.97 | 13.84 | 0.54 | 0.93 |
| AT4G16820 | lipase class 3 family protein | 28.29 | 29.07 | 1.00 | 1.05 |
| AT1G15520 | ABC transporter family protein | 22.31 | 19.64 | 0.83 | 0.79 |
| AT1G68450 | VQ motif-containing protein | 18.57 | 20.11 | 1.51 | 1.70 |
| AT2G26530 | expressed protein | 16.63 | 21.44 | 2.15 | 3.33 |
| AT4G04610 | 5'-adenylylsulfate reductase (APR1) /(PRH19) | 14.26 | 13.24 | 0.93 | 1.96 |
| AT3G54640 | tryptophan synthase, alpha subunit (TSA1) | 11.97 | 11.21 | 0.88 | 1.84 |
| AT1G05560 | UDP-glucose transferase (UGT75B2) | 10.84 | 7.26 | 0.72 | 0.64 |
| AT4G39940 | adenylylsulfate kinase 2 (AKN2) | 10.63 | 7.73 | 0.52 | 1.32 |
| AT5G57890 | anthranilate synthase beta subunit, putative | 10.02 | 8.21 | 0.91 | 1.42 |
| **Protein kinases** |  |  |  |  |  |
| AT1G74360 | leucine-rich repeat transmembrane protein kinase, putative | 15.03 | 8.90 | 0.76 | 1.36 |
| AT5G65600 | legume lectin family protein / protein kinase family protein | 11.95 | 13.27 | 0.90 | 1.11 |
| AT1G79680 | wall-associated kinase, putative | 10.21 | 9.87 | 1.06 | 0.99 |
| **Stress-induced/defense, senescence-related** | |  |  |  |  |
| AT2G15120 | pseudogene, disease-resistance family protein | 48.22 | 18.32 | 0.79 | 1.89 |
| AT1G75830 | plant defensin-fusion protein, putative (PDF1.1) | 47.09 | 1.96 | 0.04 | 0.20 |
| AT1G16030 | heat shock protein 70, putative / HSP70, putative | 45.22 | 31.35 | 0.66 | 0.93 |
| AT1G74310 | heat shock protein 101 (HSP101) | 45.03 | 23.05 | 0.88 | 0.86 |
| AT2G40170 | Em-like protein GEA6 (EM6) | 39.01 | 5.88 | 0.66 | 0.42 |
| AT2G41280 | late embryogenesis abundant protein (M10) | 31.59 | 10.29 | 0.48 | 1.20 |
| AT1G48130 | peroxiredoxin (PER1) / rehydrin, putative | 30.83 | 5.59 | 0.77 | 0.60 |
| AT1G64160 | disease resistance-responsive family protein | 27.07 | 29.12 | 1.01 | 1.38 |
| AT3G56350 | superoxide dismutase (Mn), putative | 25.63 | 9.44 | 0.69 | 0.64 |
| AT1G68850 | peroxidase, putative | 25.34 | 14.78 | 0.82 | 1.33 |
| AT1G32560 | late embryogenesis abundant group 1 domain-containing protein | 21.56 | 4.19 | 0.63 | 0.49 |
| AT5G52640 | heat shock protein 81-1 (HSP81-1) / heat shock protein 83 (HSP83) | 21.29 | 16.98 | 0.83 | 1.62 |
| AT2G15010 | thionin, putative | 20.84 | 6.00 | 0.85 | 0.59 |
| AT2G21490 | dehydrin family protein | 19.18 | 10.32 | 1.04 | 0.98 |
| AT1G02930 | glutathione S-transferase, putative | 18.64 | 8.70 | 0.59 | 2.37 |
| AT5G12020 | 17.6 kDa class II heat shock protein (HSP17.6-CII) | 18.63 | 17.92 | 0.94 | 1.32 |
| AT1G57630 | disease resistance protein (TIR class), putative | 17.38 | 18.38 | 1.04 | 1.19 |
| AT4G12400 | stress-inducible protein, putative | 15.19 | 13.48 | 0.84 | 0.84 |
| AT1G08830 | superoxide dismutase (Cu-Zn) (SODCC) | 14.90 | 1.10 | 0.09 | 0.21 |
| AT1G72900 | disease resistance protein (TIR-NBS class), putative | 13.88 | 10.68 | 0.89 | 1.09 |
| AT1G52560 | 26.5 kDa class I small heat shock protein-like (HSP26.5-P) | 10.28 | 10.47 | 1.05 | 1.10 |
| AT3G02840 | immediate-early fungal elicitor family protein | 10.18 | 11.36 | 1.00 | 1.04 |
| **Transcription** |  |  |  |  |  |
| AT4G25380 | zinc finger (AN1-like) family protein | 54.79 | 29.99 | 0.43 | 1.01 |
| AT1G18970 | germin-like protein (GLP1) (GLP4) | 36.73 | 24.91 | 0.69 | 2.25 |
| AT5G12420 | expressed protein | 26.70 | 19.97 | 0.75 | 1.85 |
| AT2G43000 | no apical meristem (NAM) family protein | 26.45 | 10.36 | 0.79 | 0.88 |
| AT5G05410 | DRE-binding protein (DREB2A) | 19.13 | 51.38 | 3.83 | 2.85 |
| AT2G40350 | AP2 domain-containing transcription factor, putative (DREB2) | 14.42 | 11.70 | 0.73 | 1.45 |
| AT4G17490 | ethylene-responsive element-binding protein, putative | 13.28 | 12.15 | 0.97 | 1.14 |
| AT2G26150 | heat shock transcription factor family protein | 13.26 | 13.45 | 0.99 | 1.21 |
| AT4G09110 | zinc finger (C3HC4-type RING finger) family protein | 13.14 | 13.47 | 1.04 | 1.25 |
| **Transporters** |  |  |  |  |  |
| AT1G04560 | AWPM-19-like membrane family protein | 34.05 | 6.85 | 1.09 | 0.88 |
| AT3G63380 | Ca(2+)-ATPase, putative (ACA12) | 24.44 | 30.95 | 1.09 | 1.93 |
| AT1G17810 | major intrinsic family protein | 19.87 | 14.64 | 1.13 | 1.15 |
| AT3G48850 | mitochondrial phosphate transporter, putative | 17.86 | 13.58 | 0.99 | 1.01 |
| AT5G13750 | transporter-related | 15.45 | 21.14 | 2.68 | 4.25 |
| AT1G71140 | MATE efflux family protein | 15.00 | 14.05 | 1.29 | 1.55 |
| AT1G73190 | alpha-TIP (TIP3.1) | 14.72 | 9.14 | 1.27 | 1.08 |
| AT2G04040 | MATE efflux family protein | 13.86 | 4.35 | 0.17 | 0.46 |
| AT1G60750 | pseudogene, aldo/keto reductase family | 11.44 | 10.87 | 1.35 | 1.20 |
| **Unknown** |  |  |  |  |  |
| AT4G37290 | expressed protein | 86.35 | 83.53 | 0.93 | 2.36 |
| AT3G16530 | legume lectin family protein | 85.76 | 63.50 | 2.09 | 4.32 |
| AT2G05880 | replication protein-related | 79.97 | 83.24 | 1.03 | 1.03 |
| AT3G28210 | zinc finger (AN1-like) family protein | 69.16 | 45.43 | 0.19 | 2.73 |
| AT1G14950 | major latex protein-related / MLP-related | 67.33 | 20.65 | 0.62 | 0.59 |
| AT3G01570 | glycine-rich protein / oleosin | 45.50 | 7.37 | 0.59 | 0.53 |
| AT3G09350 | armadillo/beta-catenin repeat family protein | 38.76 | 24.93 | 0.73 | 1.91 |
| AT5G40420 | glycine-rich protein / oleosin | 36.23 | 8.12 | 0.69 | 0.80 |
| AT1G05510 | expressed protein | 34.19 | 16.67 | 1.07 | 0.92 |
| AT2G43510 | trypsin inhibitor, putative | 32.69 | 13.93 | 0.68 | 0.53 |
| AT1G14930 | major latex protein-related / MLP-related | 32.06 | 6.58 | 0.22 | 0.65 |
| AT3G18250 | expressed protein | 29.72 | 19.49 | 0.59 | 1.45 |
| AT4G25140 | glycine-rich protein / oleosin | 27.40 | 5.67 | 0.59 | 0.53 |
| AT1G68620 | expressed protein | 22.42 | 26.06 | 2.08 | 4.32 |
| AT2G31945 | expressed protein | 22.35 | 23.01 | 1.14 | 1.14 |
| AT5G48430 | expressed protein | 22.11 | 6.18 | 0.28 | 0.47 |
| AT1G05575 | expressed protein | 21.95 | 12.71 | 1.22 | 1.21 |
| AT2G23270 | expressed protein | 21.72 | 14.77 | 0.48 | 1.10 |
| AT1G76600 | expressed protein | 20.61 | 9.94 | 0.32 | 1.26 |
| AT3G09410 | pectinacetylesterase family protein | 19.52 | 10.56 | 0.65 | 1.01 |
| AT5G45690 | expressed protein | 19.09 | 10.62 | 1.01 | 1.04 |
| AT5G42380 | calmodulin-related protein, putative | 18.52 | 19.59 | 1.13 | 1.00 |
| AT4G01870 | tolB protein-related | 15.49 | 7.80 | 0.74 | 0.98 |
| AT2G38860 | proteaseI (pfpI)-like protein (YLS5) | 14.65 | 5.29 | 0.33 | 0.55 |
| AT3G54150 | embryo-abundant protein-related | 13.92 | 12.72 | 0.99 | 1.39 |
| AT2G21820 | expressed protein | 13.49 | 5.24 | 1.08 | 0.58 |
| AT2G41730 | expressed protein | 12.35 | 0.56 | 0.03 | 0.15 |
| AT5G39670 | calcium-binding EF hand family protein | 11.03 | 12.64 | 0.97 | 1.49 |
| AT5G64510 | expressed protein | 10.28 | 16.99 | 1.57 | 1.98 |

a: MFIB (w/c): Mean fold induction in gene expression between *WT* and *cry1* in blue light;

b: MFI_*WT* (B/D): Mean fold induction in gene expression between blue light and the dark in *WT*;

c: MFI_*cry1* (B/D): Mean fold induction in gene expression between blue light and the dark in *cry1* mutants;

d: MFI_*hfr1* (B/D): Mean fold induction in gene expression between blue light and the dark in *hfr1* mutants.
