# Supplementary material for: HFR1 Is Crucial for Transcriptome Regulation in the Cryptochrome 1-Mediated Early Response to Blue Light in Arabidopsis thaliana
Source: PLoS One. 2008 Oct 30;3(10):e3563. doi: 10.1371/journal.pone.0003563 (PMC2570330; doi:10.1371/journal.pone.0003563)
Supplement: Data S4 — Genes induced by blue light in cry1- and HFR1-dependent manner with 10>MFI> = 5. (0.19 MB DOC) [file pone.0003563.s004.doc]

**Data S4 Genes induced by blue light in cry1- and HFR1-dependent manner with 10>MFI≥5.**

| **AGI Locus** | **Gene Description** | **^a^MFIB(w/c)** | **^b^MFI_*WT*(B/D)** | **^c^MFI_*cry1*(B/D)** | **^d^MFI_*hfr1*(B/D)** |
| --- | --- | --- | --- | --- | --- |
| **Electron transport** | |  |  |  |  |
| AT3G26200 | cytochrome P450 71B22, putative (CYP71B22) | 9.46 | 9.82 | 1.02 | 1.12 |
| AT4G20830 | FAD-binding domain-containing protein | 6.38 | 8.25 | 1.40 | 2.06 |
| AT4G37310 | cytochrome P450, putative (CYP81H1) | 5.24 | 5.13 | 1.07 | 1.07 |
| **Hormone-related** | |  |  |  |  |
| AT3G13610 | oxidoreductase, 2OG-Fe(II) oxygenase family protein | 8.10 | 4.41 | 0.30 | 1.26 |
| **Metabolism** |  |  |  |  |  |
| AT3G53160 | UDP-glucoronosyl/UDP-glucosyl transferase family protein | 9.26 | 8.24 | 1.12 | 1.29 |
| AT3G23570 | dienelactone hydrolase family protein | 8.04 | 4.34 | 0.51 | 1.10 |
| AT2G43570 | chitinase, putative | 8.03 | 9.67 | 1.74 | 2.54 |
| AT2G02010 | glutamate decarboxylase, putative | 7.89 | 10.37 | 1.21 | 1.48 |
| AT4G11280 | ACC synthase 6 (ACS6) | 7.87 | 6.86 | 1.77 | 0.74 |
| AT1G55850 | cellulose synthase family protein | 7.61 | 5.16 | 0.70 | 1.09 |
| AT1G33030 | O-methyltransferase family 2 protein | 7.45 | 5.75 | 0.72 | 1.33 |
| AT5G16970 | NADP-dependent oxidoreductase, putative (P1) | 7.28 | 11.03 | 1.47 | 1.98 |
| AT5G58840 | subtilase family protein | 7.23 | 7.97 | 0.95 | 1.04 |
| AT4G34135 | UDP-glucoronosyl/UDP-glucosyl transferase family protein | 6.66 | 4.16 | 0.82 | 1.11 |
| AT2G29420 | glutathione S-transferase, putative | 6.54 | 5.22 | 0.85 | 1.07 |
| AT4G26270 | phosphofructokinase family protein | 6.46 | 3.15 | 0.41 | 0.44 |
| AT4G28420 | aminotransferase, putative | 5.15 | 4.37 | 0.71 | 1.11 |
| **Photosysthesis/chloroplast proteins** | |  |  |  |  |
| AT2G38470 | WRKY family transcription factor | 9.27 | 8.26 | 1.11 | 1.28 |
| AT2G04400 | indole-3-glycerol phosphate synthase (IGPS) | 7.40 | 4.26 | 0.65 | 0.92 |
| AT4G27070 | tryptophan synthase, beta subunit 2 (TSB2) | 7.34 | 9.17 | 1.35 | 1.42 |
| AT4G37710 | VQ motif-containing protein | 6.13 | 6.45 | 1.05 | 1.10 |
| AT4G36040 | DNAJ heat shock N-terminal domain-containing protein (J11) | 6.04 | 5.47 | 1.35 | 1.19 |
| AT2G25735 | expressed protein | 5.98 | 4.20 | 0.82 | 0.94 |
| AT4G21990 | 5'-adenylylsulfate reductase (APR3) / (PRH26) | 5.92 | 7.48 | 0.96 | 2.88 |
| AT3G08760 | protein kinase family protein | 5.81 | 7.05 | 1.29 | 1.38 |
| AT2G32210 | expressed protein | 5.57 | 3.66 | 0.43 | 1.19 |
| AT1G08050 | zinc finger (C3HC4-type RING finger) family protein | 5.40 | 3.58 | 0.79 | 1.04 |
| AT1G52200 | expressed protein | 5.04 | 3.60 | 1.44 | 0.82 |
| **Protein kinases** | |  |  |  |  |
| AT4G18950 | ankyrin protein kinase, putative | 9.78 | 10.61 | 1.37 | 2.24 |
| AT5G25930 | leucine-rich repeat family protein / protein kinase family protein | 9.08 | 8.16 | 1.25 | 1.34 |
| AT1G01560 | mitogen-activated protein kinase, putative / MAPK, putative (MPK11) | 6.18 | 6.22 | 0.95 | 1.09 |
| AT1G66880 | serine/threonine protein kinase family protein | 5.92 | 1.99 | 0.43 | 0.29 |
| AT1G63560 | receptor-like protein kinase-related | 5.25 | 5.37 | 1.04 | 1.09 |
| **Stress-induced/defense, senescence-related** | |  |  |  |  |
| AT5G51440 | 23.5 kDa mitochondrial small heat shock protein (HSP23.5-M) | 9.97 | 2.22 | 0.13 | 0.49 |
| AT1G14550 | anionic peroxidase, putative | 9.55 | 5.44 | 0.90 | 1.18 |
| AT2G35300 | late embryogenesis abundant group 1 domain-containing protein | 8.15 | 2.71 | 0.70 | 0.89 |
| AT4G30530 | defense-related protein, putative | 7.87 | 5.94 | 0.76 | 0.77 |
| AT2G20560 | DNAJ heat shock family protein | 7.76 | 6.25 | 1.01 | 1.16 |
| AT3G12580 | heat shock protein 70, putative / HSP70, putative | 7.06 | 8.83 | 1.63 | 1.18 |
| AT3G22500 | late embryogenesis abundant protein (ECP31) | 6.66 | 5.51 | 1.23 | 1.00 |
| AT2G32120 | heat shock protein 70 family protein / HSP70 family protein | 5.74 | 4.56 | 0.95 | 0.92 |
| AT5G39580 | peroxidase, putative | 5.60 | 6.24 | 1.16 | 1.36 |
| AT4G08770 | peroxidase, putative | 5.47 | 3.43 | 0.52 | 0.93 |
| **Transcription** |  |  |  |  |  |
| AT1G02220 | no apical meristem (NAM) family protein | 9.89 | 9.33 | 0.77 | 1.87 |
| AT1G18570 | myb family transcription factor (MYB51) | 9.29 | 9.82 | 2.04 | 1.33 |
| AT3G24500 | ethylene-responsive transcriptional coactivator, putative | 8.37 | 11.01 | 2.39 | 1.16 |
| AT3G50260 | AP2 domain-containing transcription factor, putative | 6.62 | 3.55 | 0.62 | 0.77 |
| AT1G62300 | WRKY family transcription factor | 6.18 | 8.51 | 1.19 | 2.05 |
| AT2G24500 | zinc finger (C2H2 type) family protein | 5.98 | 4.27 | 0.59 | 0.83 |
| AT3G23250 | myb family transcription factor (MYB15) | 5.78 | 5.43 | 0.82 | 1.07 |
| **Transporters** |  |  |  |  |  |
| AT2G29470 | glutathione S-transferase, putative | 7.31 | 7.67 | 1.09 | 1.09 |
| AT1G60730 | aldo/keto reductase family protein | 5.88 | 4.28 | 0.70 | 0.60 |
| AT3G25610 | haloacid dehalogenase-like hydrolase family protein | 5.70 | 3.27 | 0.63 | 1.02 |
| AT5G64250 | 2-nitropropane dioxygenase family / NPD family | 5.60 | 6.30 | 2.21 | 1.86 |
| AT1G12200 | flavin-containing monooxygenase family protein | 5.56 | 4.09 | 0.65 | 0.80 |
| AT5G01670 | aldose reductase, putative | 5.48 | 3.93 | 0.90 | 0.44 |
| AT1G01340 | cyclic nucleotide-regulated ion channel (CNGC10) (ACBK1) | 5.16 | 6.73 | 1.31 | 1.23 |
| **Unknown** |  |  |  |  |  |
| AT2G41100 | touch-responsive protein / calmodulin-related protein 3 | 9.87 | 7.44 | 1.11 | 0.64 |
| AT2G35980 | harpin-induced family protein (YLS9) / HIN1 family protein | 9.80 | 8.39 | 2.70 | 2.12 |
| AT5G10695 | expressed protein | 9.50 | 3.05 | 0.47 | 0.38 |
| AT5G14730 | expressed protein | 9.07 | 4.17 | 0.31 | 0.71 |
| AT1G28190 | expressed protein | 9.02 | 6.57 | 1.02 | 1.05 |
| AT4G28460 | hypothetical protein | 8.55 | 5.40 | 0.48 | 1.27 |
| AT4G11370 | zinc finger (C3HC4-type RING finger) family protein | 8.39 | 10.64 | 1.19 | 1.22 |
| AT1G19020 | expressed protein | 8.15 | 3.69 | 1.01 | 0.56 |
| AT1G69890 | expressed protein | 7.72 | 9.64 | 1.23 | 1.24 |
| AT5G25260 | expressed protein | 7.50 | 9.07 | 1.46 | 1.50 |
| AT5G37840 | expressed protein | 7.14 | 7.20 | 0.93 | 0.99 |
| AT4G26120 | BTB/POZ domain-containing protein | 7.00 | 7.38 | 1.01 | 0.90 |
| AT4G31830 | expressed protein | 6.80 | 5.64 | 0.93 | 1.10 |
| AT2G46650 | cytochrome b5, putative | 6.79 | 3.83 | 0.27 | 0.53 |
| AT4G39670 | expressed protein | 6.57 | 6.64 | 1.07 | 2.35 |
| AT3G48450 | nitrate-responsive NOI protein, putative | 6.53 | 5.61 | 1.00 | 1.43 |
| AT5G07330 | expressed protein | 6.19 | 3.15 | 1.04 | 1.00 |
| AT1G05060 | expressed protein | 6.08 | 5.45 | 0.65 | 1.02 |
| AT1G13340 | expressed protein | 6.06 | 4.93 | 0.53 | 0.76 |
| AT5G27760 | hypoxia-responsive family protein | 5.88 | 4.23 | 0.79 | 1.24 |
| AT1G55450 | embryo-abundant protein-related | 5.57 | 4.06 | 1.11 | 0.87 |
| AT5G09800 | U-box domain-containing protein | 5.55 | 5.06 | 0.59 | 1.33 |
| AT1G25400 | expressed protein | 5.53 | 2.44 | 0.46 | 0.78 |
| AT3G28930 | avrRpt2-induced AIG2 protein (AIG2) | 5.50 | 6.60 | 1.42 | 1.02 |
| AT1G55920 | serine O-acetyltransferase, putative | 5.40 | 4.30 | 1.17 | 0.97 |
| AT2G18680 | expressed protein | 5.24 | 5.31 | 0.97 | 1.03 |
| AT5G25450 | ubiquinol-cytochrome C reductase complex 14 kDa protein, putative | 5.19 | 6.52 | 1.28 | 1.95 |

a: MFIB (w/c): Mean fold induction in gene expression between *WT* and *cry1* in blue light;

b: MFI_*WT* (B/D): Mean fold induction in gene expression between blue light and the dark in *WT*;

c: MFI_*cry1* (B/D): Mean fold induction in gene expression between blue light and the dark in *cry1* mutants;

d: MFI_*hfr1* (B/D): Mean fold induction in gene expression between blue light and the dark in *hfr1* mutants.
