# Supplementary material for: HFR1 Is Crucial for Transcriptome Regulation in the Cryptochrome 1-Mediated Early Response to Blue Light in Arabidopsis thaliana
Source: PLoS One. 2008 Oct 30;3(10):e3563. doi: 10.1371/journal.pone.0003563 (PMC2570330; doi:10.1371/journal.pone.0003563)
Supplement: Data S5 — Genes induced by blue light in cry1- and HFR1-dependent manner with 5>MFI> = 2. (0.15 MB DOC) [file pone.0003563.s005.doc]

**Data S5 Genes induced by blue light in cry1- and HFR1-dependent manner with 5>MFI≥2.**

| **AGI Locus** | **Gene Description** | **^a^MFIB(w/c)** | **^b^MFI_*WT*(B/D)** | **^c^MFI_*cry1*(B/D)** | **^d^MFI_*hfr1*(B/D)** |
| --- | --- | --- | --- | --- | --- |
| **Electron transport** | |  |  |  |  |
| AT4G18360 | (S)-2-hydroxy-acid oxidase, peroxisomal, putative | 3.03 | 2.42 | 1.07 | 1.03 |
| AT3G26220 | cytochrome P450 family protein (CYP71B3) | 2.67 | 3.18 | 1.21 | 0.99 |
| **Growth and development related proteins** | |  |  |  |  |
| AT1G66580 | 60S ribosomal protein L10 (RPL10C) | 4.37 | 3.57 | 0.64 | 1.32 |
| AT1G25275 | expressed protein | 3.88 | 1.46 | 0.45 | 0.34 |
| AT1G55530 | zinc finger (C3HC4-type RING finger) family protein | 2.41 | 2.89 | 1.09 | 0.70 |
| AT3G08690 | ubiquitin-conjugating enzyme 11 | 2.08 | 2.20 | 0.88 | 1.00 |
| **Metabolism** |  |  |  |  |  |
| AT4G24160 | hydrolase, alpha/beta fold family protein | 4.75 | 3.60 | 0.94 | 1.49 |
| AT2G23540 | GDSL-motif lipase/hydrolase family protein | 4.73 | 1.79 | 0.39 | 0.47 |
| AT2G30140 | UDP-glucoronosyl/UDP-glucosyl transferase family protein | 4.43 | 5.45 | 0.85 | 1.35 |
| AT2G47180 | galactinol synthase, putative | 4.34 | 4.39 | 1.51 | 0.93 |
| AT5G20960 | aldehyde oxidase 1 (AAO1) | 3.91 | 4.45 | 1.91 | 0.86 |
| AT1G01480 | ACC synthase 2 (ACS2) (ACC1) | 3.79 | 2.47 | 0.72 | 0.48 |
| AT1G14130 | 2-oxoglutarate-dependent dioxygenase, putative | 3.78 | 4.63 | 1.46 | 1.43 |
| AT2G29490 | glutathione S-transferase, putative | 3.71 | 3.77 | 1.27 | 1.54 |
| AT4G13180 | short-chain dehydrogenase/reductase (SDR) family protein | 3.41 | 2.46 | 0.51 | 1.01 |
| AT2G19450 | diacylglycerol O-acyltransferase | 3.34 | 2.11 | 0.55 | 0.83 |
| AT5G23510 | expressed protein | 3.27 | 2.88 | 1.09 | 0.87 |
| AT5G40010 | AAA-type ATPase family protein | 2.95 | 3.32 | 0.64 | 1.11 |
| AT4G30490 | AFG1-like ATPase family protein | 2.60 | 2.49 | 1.00 | 1.12 |
| AT5G17380 | pyruvate decarboxylase family protein | 2.17 | 1.66 | 0.83 | 0.76 |
| AT4G10520 | subtilase family protein | 2.09 | 2.17 | 1.03 | 1.05 |
| **Photosysthesis/chloroplast proteins** | |  |  |  |  |
| AT2G38340 | AP2 domain-containing transcription factor, putative (DRE2B) | 4.95 | 4.45 | 0.40 | 1.06 |
| AT5G26920 | calmodulin-binding protein | 4.30 | 4.91 | 1.11 | 0.93 |
| AT3G22890 | sulfate adenylyltransferase 1 / ATP-sulfurylase 1 (APS1) | 4.19 | 3.90 | 0.91 | 1.37 |
| AT2G19310 | expressed protein | 3.68 | 4.29 | 1.20 | 1.14 |
| AT1G67810 | Fe-S metabolism associated domain-containing protein | 3.45 | 5.91 | 0.97 | 0.90 |
| AT1G76070 | expressed protein | 3.33 | 4.49 | 1.33 | 1.08 |
| AT1G36370 | glycine hydroxymethyltransferase | 3.25 | 2.77 | 0.73 | 1.25 |
| AT3G44720 | prephenate dehydratase family protein | 2.61 | 10.31 | 3.21 | 2.45 |
| AT4G30210 | NADPH-cytochrome p450 reductase | 2.26 | 2.03 | 0.73 | 0.49 |
| **Stress-induced/defense, senescence-related** | |  |  |  |  |
| AT5G05340 | peroxidase, putative | 4.66 | 4.78 | 0.88 | 1.08 |
| AT4G19880 | glutathione S-transferase-related | 4.00 | 3.70 | 1.43 | 1.27 |
| AT4G15420 | PRLI-interacting factor K | 3.98 | 3.21 | 0.65 | 1.06 |
| AT2G23810 | senescence-associated family protein | 3.73 | 3.70 | 1.62 | 1.33 |
| AT1G12520 | superoxide dismutase copper chaperone, putative | 3.69 | 0.52 | 0.13 | 0.12 |
| AT2G47730 | glutathione S-transferase 6 (GST6) | 3.40 | 4.50 | 1.27 | 2.30 |
| AT1G78380 | glutathione S-transferase, putative | 2.63 | 4.76 | 1.90 | 1.96 |
| **Transcription** |  |  |  |  |  |
| AT1G22985 | AP2 domain-containing transcription factor, putative | 4.95 | 2.12 | 0.42 | 0.45 |
| AT3G53230 | cell division cycle protein 48, putative | 4.77 | 3.40 | 0.40 | 0.89 |
| AT2G23320 | WRKY family transcription factor | 4.57 | 3.28 | 0.53 | 0.84 |
| AT3G10500 | no apical meristem (NAM) family | 4.32 | 3.05 | 0.70 | 1.01 |
| AT5G24110 | WRKY family transcription factor | 2.02 | 2.17 | 1.04 | 1.06 |
| **Transporters** |  |  |  |  |  |
| AT1G30400 | glutathione S-conjugate ABC transporter | 4.60 | 5.84 | 1.77 | 1.45 |
| AT1G33110 | MATE efflux family protein | 4.54 | 6.10 | 1.20 | 1.27 |
| AT4G21680 | proton-dependent oligopeptide transport (POT) family protein | 4.47 | 5.21 | 1.01 | 1.49 |
| AT1G75270 | dehydroascorbate reductase, putative | 4.19 | 5.82 | 1.34 | 2.53 |
| AT4G28390 | ADP, ATP carrier protein, putative | 3.25 | 2.01 | 0.54 | 0.61 |
| AT3G47730 | ABC transporter family protein | 2.96 | 2.40 | 0.81 | 0.74 |
| AT2G34660 | glutathione S-conjugate ABC transporter (MRP2) | 2.90 | 5.73 | 2.08 | 2.41 |
| AT3G09270 | glutathione S-transferase, putative | 2.82 | 1.96 | 0.81 | 1.12 |
| AT3G18830 | mannitol transporter, putative | 2.78 | 2.55 | 0.84 | 0.72 |
| **Unknown** |  |  |  |  |  |
| AT3G61390 | U-box domain-containing protein | 4.71 | 4.68 | 1.11 | 1.20 |
| AT3G52450 | U-box domain-containing protein | 4.43 | 4.46 | 0.84 | 1.23 |
| AT3G61190 | BON1-associated protein 1 (BAP1) | 4.16 | 4.24 | 0.85 | 1.05 |
| AT3G12960 | expressed protein | 4.06 | 4.86 | 1.20 | 1.16 |
| AT3G48890 | cytochrome b5 domain-containing protein | 3.94 | 4.79 | 1.29 | 1.54 |
| AT3G26910 | hydroxyproline-rich glycoprotein | 3.88 | 3.17 | 0.91 | 1.05 |
| AT1G16730 | expressed protein | 3.39 | 3.41 | 1.02 | 1.03 |
| AT2G16900 | expressed protein | 3.22 | 4.27 | 1.29 | 1.25 |
| AT1G15430 | expressed protein | 2.92 | 3.72 | 1.07 | 1.26 |
| AT5G66440 | expressed protein | 2.30 | 2.95 | 1.26 | 0.91 |
| At1g23430 | hypothetical protein | 2.20 | 1.66 | 0.81 | 0.63 |

a: MFIB (w/c): Mean fold induction in gene expression between *WT* and *cry1* in blue light;

b: MFI_*WT* (B/D): Mean fold induction in gene expression between blue light and the dark in *WT*;

c: MFI_*cry1* (B/D): Mean fold induction in gene expression between blue light and the dark in *cry1* mutants;

d: MFI_*hfr1* (B/D): Mean fold induction in gene expression between blue light and the dark in *hfr1* mutants.
