# Supplementary material for: HFR1 Is Crucial for Transcriptome Regulation in the Cryptochrome 1-Mediated Early Response to Blue Light in Arabidopsis thaliana
Source: PLoS One. 2008 Oct 30;3(10):e3563. doi: 10.1371/journal.pone.0003563 (PMC2570330; doi:10.1371/journal.pone.0003563)
Supplement: Data S6 — Genes repressed by blue light in cry1- and HFR1-dependent manner. (0.12 MB DOC) [file pone.0003563.s006.doc]

**Data S6 Genes repressed by blue light in cry1- and HFR1-dependent manner.**

| **AGI Locus** | **Gene Description** | **^a^MFRB(c/w)** | **^b^MFR_*WT*(D/B)** | **^c^MFR_*cry1*(D/B)** | **^d^MFR_*hfr1*(D/B)** |
| --- | --- | --- | --- | --- | --- |
| **Electron transport** | |  |  |  |  |
| AT2G39510 | nodulin MtN21 family protein | 8.09 | 37.32 | 3.78 | 3.77 |
| AT4G01430 | nodulin MtN21 family protein | 4.34 | 3.16 | 0.34 | 0.61 |
| AT1G12740 | cytochrome P450 family protein (CYP87A2) | 4.22 | 3.63 | 0.59 | 0.48 |
| AT2G42850 | cytochrome P450 family protein (CYP718) | 3.83 | 11.54 | 1.77 | 1.93 |
| AT1G67110 | cytochrome P450, putative (CYP735A2) | 2.79 | 3.04 | 0.56 | 0.70 |
| **Growth/development** | |  |  |  |  |
| AT2G33790 | pollen Ole e 1 allergen and extensin family protein | 5.62 | 2.04 | 0.37 | 0.11 |
| AT2G41830 | cyclin-related | 2.39 | 2.61 | 0.88 | 0.88 |
| AT2G13820 | protease inhibitor/seed storage | 2.21 | 2.21 | 1.07 | 1.01 |
| **Metabolism** |  |  |  |  |  |
| AT5G48880 | acetyl-CoA C-acyltransferase 1 | 9.22 | 0.97 | 0.15 | 0.10 |
| AT5G04960 | pectinesterase family protein | 6.89 | 11.86 | 1.86 | 0.46 |
| AT4G15400 | transferase family protein | 3.19 | 3.58 | 0.70 | 0.77 |
| AT4G26220 | caffeoyl-CoA 3-O-methyltransferase, putative | 3.11 | 5.96 | 0.95 | 1.10 |
| AT5G47950 | transferase family protein | 2.90 | 7.31 | 1.48 | 1.93 |
| AT1G31950 | terpene synthase/cyclase family protein | 2.83 | 2.79 | 0.64 | 0.78 |
| AT2G23620 | esterase, putative | 2.53 | 5.76 | 1.71 | 1.18 |
| AT3G11950 | UbiA prenyltransferase family protein | 2.53 | 2.58 | 1.15 | 0.99 |
| AT4G21850 | SeIR domain-containing protein | 2.52 | 3.09 | 0.88 | 1.06 |
| AT3G01260 | aldose 1-epimerase family protein | 2.20 | 1.41 | 0.38 | 0.51 |
| AT5G04950 | nicotianamine synthase, putative | 2.13 | 1.38 | 0.34 | 0.34 |
| AT5G52790 | CBS domain-containing protein-related | 2.06 | 2.75 | 0.27 | 0.43 |
| **Photosynthesis/chloroplast proteins** | | |  |  |  |
| AT2G21385 | expressed protein | 3.41 | 2.68 | 0.79 | 1.07 |
| AT5G52540 | expressed protein | 2.05 | 2.14 | 1.00 | 0.85 |
| **Stress-induced/defense, senescence-related** | | |  |  |  |
| AT2G39040 | peroxidase, putative | 4.54 | 2.72 | 0.43 | 0.28 |
| AT4G26010 | peroxidase, putative | 3.59 | 5.33 | 1.40 | 0.37 |
| AT2G19970 | pathogenesis-related protein, putative | 2.36 | 4.77 | 1.11 | 1.47 |
| AT2G39430 | disease resistance-responsive protein-related | 2.06 | 2.35 | 0.91 | 0.66 |
| **Transcription** |  |  |  |  |  |
| AT1G01060 | myb family transcription factor | 2.74 | 0.45 | 0.12 | 0.07 |
| AT2G46830 | myb-related transcription factor (CCA1) | 2.60 | 0.37 | 0.10 | 0.05 |
| AT5G54230 | myb family transcription factor (MYB49) | 2.39 | 0.62 | 0.08 | 0.08 |
| AT1G72200 | zinc finger (C3HC4-type RING finger) family protein | 2.02 | 1.52 | 0.32 | 0.51 |
| **Transporters** |  |  |  |  |  |
| AT5G62210 | embryo-specific protein-related | 14.54 | 1.45 | 0.15 | 0.20 |
| AT5G62340 | invertase | 4.62 | 1.87 | 0.26 | 0.15 |
| AT1G32450 | POT family protein | 3.68 | 2.20 | 0.31 | 0.79 |
| AT1G54970 | proline-rich family protein | 3.65 | 8.26 | 1.11 | 0.23 |
| AT5G47450 | major intrinsic family protein | 2.98 | 1.75 | 0.12 | 0.28 |
| AT5G59520 | zinc transporter (ZIP2) | 2.46 | 1.36 | 0.35 | 0.52 |
| AT1G22500 | zinc finger (C3HC4-type RING finger) family protein | 2.31 | 2.02 | 0.51 | 0.73 |
| **Unknown** |  |  |  |  |  |
| AT3G29780 | expressed protein | 3.41 | 7.49 | 1.06 | 1.27 |
| AT1G30260 | expressed protein | 3.25 | 1.24 | 0.13 | 0.23 |
| AT4G26320 | arabinogalactan-protein (AGP13) | 2.27 | 5.22 | 2.02 | 2.27 |
| AT5G56540 | arabinogalactan-protein (AGP14) | 2.17 | 3.58 | 1.13 | 1.28 |

a: MFRB (w/c): Mean fold reduction in gene expression between *WT* and *cry1* in blue light;

b: MFR_*WT* (B/D): Mean fold reduction in gene expression between blue light and the dark in *WT*;

c: MFR_*cry1* (B/D): Mean fold reduction in gene expression between blue light and the dark in *cry1* mutants;

d: MFR_*hfr1* (B/D): Mean fold reduction in gene expression between blue light and the dark in *hfr1* mutants.
