# Supplementary material for: HFR1 Is Crucial for Transcriptome Regulation in the Cryptochrome 1-Mediated Early Response to Blue Light in Arabidopsis thaliana
Source: PLoS One. 2008 Oct 30;3(10):e3563. doi: 10.1371/journal.pone.0003563 (PMC2570330; doi:10.1371/journal.pone.0003563)
Supplement: Data S7 — Primers for PCR amplification (0.02 MB DOC) [file pone.0003563.s007.doc]

**Data S7 Primers for PCR amplification.**

# USE REFERENCE AGI_LOCUS SEQUENCE FROM 5’ TO 3’

# Mapping AP1 GTAATACGACTCACTCACTATCGGGC

**AP2** ACTATAGGGCACGCGTGGT

# Genotyping HFR1-F1 At1g02340 GATTACTCTAAAGTCGATATATGC

**HFR1-R1 At1g02340** AAGGAACTTCTCTTGTAAACTCCTC

**HFR1-R2 At1g02340** CCTCCTGTTTAATTATACTTACAT

**PCGN1547L-3 for *hfr1*** CGCCTATAAATACGACGGATCGTAA

**PCGN1547L-5 for *hfr1*** TAATAACGCTGCGGACATCTA

**RT-PCR CYP82C2-F At4g31970** GCACCAAGTGGTGCGTGGCCC

## CYP82C2-R At4g31970 CTTCTTCCCGAACCAAATGGC

**CYP71A12-F At2G30750** GGAGATGATATTGATGGTCTCT

**CYP71A12-R At2G30750** CCGAATCTCGTCTTGGAGTTTCTT

**CYP71B15-F At3g26830** CCCATCATCGGAAACTTACACC

**CYP71B15-R At3g26830** GCCAGCGACTCCACCAATCCC

**HSP17.4-F At3g46230** CGTGTTCGACCCATTTTCAC

**HSP17.4-R At3g46230** GGACTTGACCTCCGGCTTAC

**HSP17.6-F At1g59860** CGGCAACAACAGGCGAATCAAC

**HSP17.6-R At1g59860** CACAGTCAAAACACCATTCTCC

**CYP79B2-F At4g39950** CCACCATTAAGGAGCTTGTAA

**CYP79B2-R At4g39950** CGTCGGGTAGAGATGCTCCGGC

**CYP706A2-F At4g22710** GATGAGAAAGCTCCTCTGTCC

**CYP706A2-R At4g22710** CACCTCCATTGAAATCAAGCG

**GST-F At1g69930** GATACTTTGAGTTCGGAGAGCG

**GST-R At1g69930** CAGGCTTGACCGCTTCATGGGC

**WRKY53-F At4g23810** GCTTCAGGCACGACTTAGAGAA

**WRKY53-R At4g23810** GGCGTATCAGGGAACGAGAA

**GAPDH-F At1g13440** CCTTTCATCACCACCGAGTACATG

**GAPDH-R At1g13440** CAACCACACACAAACTCTCGCCG
